# Association between oral JAK-1 inhibitors and infection risks in atopic dermatitis: a retrospective analysis of the FAERS database

**DOI:** 10.3389/fmed.2025.1694688

**Published:** 2025-11-03

**Authors:** Zenan Tang, Zhanglei Mu, Xiaojie Wang, Yan Zhao

**Affiliations:** Department of Dermatology, Peking University People’s Hospital, Beijing, China

**Keywords:** Janus kinase inhibitors, atopic dermatitis, adverse events, FAERS database, infection

## Abstract

**Background:**

Janus kinase (JAK)-1 inhibitors have been approved for moderate-to-severe atopic dermatitis (AD). Despite favorable efficacy, their real-world infection risk profile requires further investigation.

**Methods:**

We conducted a retrospective disproportionality analysis using the U.S. Food and Drug Administration Adverse Event Reporting System (FAERS) database. Reports identifying upadacitinib or abrocitinib as primary suspect drugs for “Infections and Infestations” adverse events (AEs) in AD treatment from Q3 2019 to Q1 2025 were included. Four disproportionality methods were employed to detect infection-related safety signals.

**Results:**

A total of 18 infection-related positive safety signals associated with abrocitinib were identified, which include known AEs (herpes zoster, eczema herpeticum, and herpes simplex) and unexpected signals (sepsis, appendicitis, and septic shock). Upadacitinib showed 64 infection-related signals, encompassing known AEs (herpes zoster, pneumonia, and influenza) and unexpected signals (sepsis, appendicitis, and septic shock). Herpes zoster was the most frequent infection-related AE for both drugs.

**Conclusion:**

This study confirms established infection risks of JAK-1 inhibitors in AD (particularly herpes zoster) and identifies novel potential safety signals (sepsis, appendicitis, and septic shock). These findings provide real-world insights into the risk of infections associated with JAK inhibitors.

## 1 Introduction

Atopic dermatitis (AD) is a common chronic inflammatory skin disease, affecting approximately 10% of adults and 15–20% of children globally (1). It is characterized by persistent pruritus, skin lesions, and recurrent flares, all of which significantly impair the quality of life and social functioning (2, 3). Conventional systemic treatments, including immunosuppressants such as cyclosporine and methotrexate, often fail to meet the long-term needs of patients with moderate to severe AD (4). In recent years, Janus kinase (JAK) inhibitors have shown excellent efficacy for AD. Upadacitinib and abrocitinib are the two JAK-1 inhibitors currently approved by the FDA for the treatment of moderate-to-severe AD. They block the signaling of multiple key inflammatory cytokines, including IL-4, IL-13, IL-31, and TSLP (5, 6), thereby providing new therapeutic options for patients with AD (7, 8).

Despite the overall favorable tolerability and perceived safety of JAK-1 inhibitors, immunosuppressive effects may increase the risk of adverse events (AEs), particularly infections (9). Tsai et al. (10) reported a higher incidence of skin infections, herpes simplex, and herpes zoster with oral JAK inhibitors compared to dupilumab in the treatment of AD. Furthermore, a meta-analysis of 18 randomized controlled trials (RCTs) demonstrated that both abrocitinib and upadacitinib significantly increased the risk of infections compared to placebo (11). Another study involving 1,793 AD patients (aged ≥12 years) also demonstrated a higher frequency of infections, notably herpesvirus infections, with JAK inhibitors relative to dupilumab (12). However, the relatively short follow-up duration in the meta-analysis (generally no longer than 12 months) makes it difficult to detect rare safety events. Additionally, the strict eligibility criteria in clinical trials often exclude special populations, including the elderly and individuals with comorbidities, thereby limiting the generalizability of the reported safety outcomes to real-world settings.

The U.S. Food and Drug Administration Adverse Event Reporting System (FAERS) is a comprehensive database overseen by the U.S. Food and Drug Administration, which collects information on AEs and medication errors (13, 14). Due to its public accessibility and large volume of data, the FAERS database has been increasingly utilized by researchers to investigate the safety profiles of drugs in the real world (15, 16). This study used the FAERS database to investigate the association between oral JAK-1 inhibitors used for the treatment of AD and the risk of infection-related adverse events.

## 2 Methods

### 2.1 Data source

**Figure 1:**
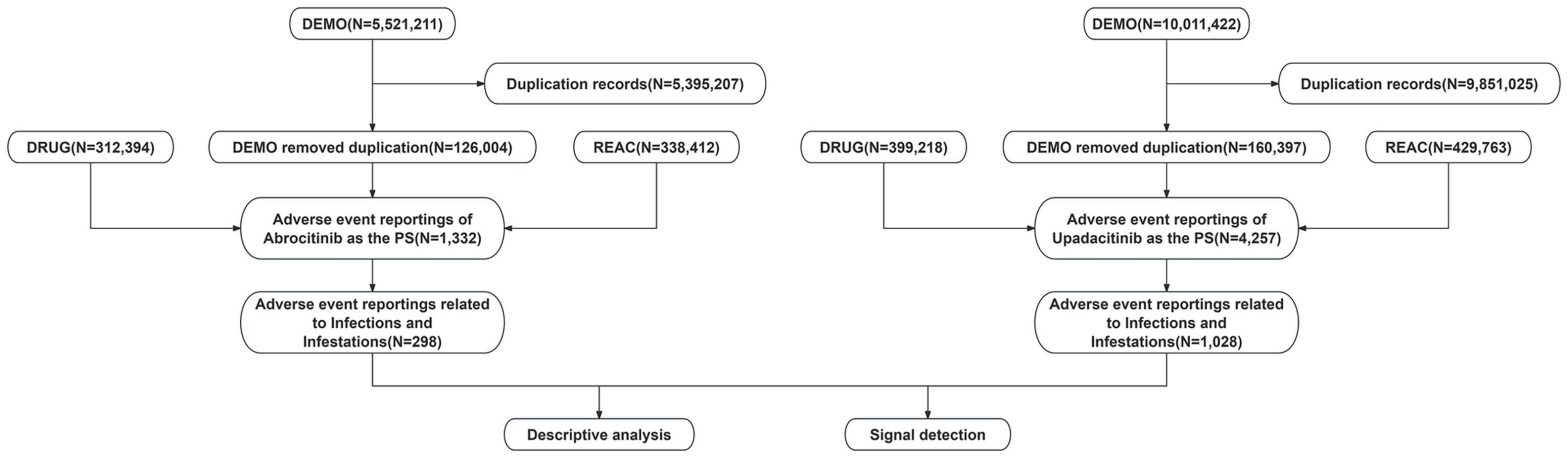
Flowchart using the FAERS database.

The data were sourced from the publicly available FAERS database, accessible at https://fis.fda.gov/extensions/FPD-QDE-FAERS/FPDQDE-FAERS.html. The database consists of spontaneous, anonymized reports of adverse events submitted voluntarily by healthcare professionals and the public. As all data are de-identified and contain no personally identifiable information, and since the use of this database does not involve direct interaction with human subjects, the approval of the ethics committee approval was not required. As a critical resource in pharmacovigilance, the FAERS database consists of seven core data components: DEMO (demographics), DRUG (drug-related information), REAC (AEs), OUTC (outcomes), RPSR (report sources), THER (therapy initiation and cessation dates), and INDI (indications). This retrospective analysis focused on adverse event reports in which upadacitinib or abrocitinib were identified as the primary suspect drug, including reports from the third quarter of 2019 (Q3 2019) to the first quarter of 2025 (Q1 2025). A detailed flowchart outlining the data extraction, processing, and analytical procedures is provided in Figure 1.

### 2.2 Data processing

Data deduplication was performed in accordance with the FDA recommendations by removing duplicate reports based on case identifiers (CASEIDs), FDA receipt date (FDA_DT), and primary identifier (PRIMARYID). Specifically, for reports sharing the same CASEID, the entry with the most recent FDA_DT was retained. In cases where both CASEID and FDA_DT were identical, the record with the highest PRIMARYID was selected. Following deduplication, adverse event terms were standardized using the Medical Dictionary for Regulatory Activities (MedDRA version 27.1) and classified at both the System Organ Class (SOC) and Preferred Term (PT) levels. To identify relevant cases, the SOC field was queried for the term “Infections and Infestations.” Subsequently, among these, reports indicating AD as the indication were extracted and considered to involve the primary suspect drugs.

### 2.3 Data mining

During the data mining phase, this study utilized multiple disproportionality analysis methods, including the Reporting Odds Ratio (ROR), the Proportional Reporting Ratio (PRR), the Bayesian Confidence Propagation Neural Network (BCPNN), and the Empirical Bayesian Geometric Mean (EBGM). Disproportionality analysis is based on the classic fourfold contingency table (Table 1). The values for each metric of ROR, PRR, BCPNN, and EBGM were calculated according to their specific formulas (Table 2). Elevated values indicate more robust signals of AEs associated with JAK inhibitors, reflecting a stronger statistical correlation between these drugs and specific AEs. By integrating the four algorithms, we aimed to leverage their respective strengths, cross-validate findings from multiple perspectives, and identify additional potential rare adverse reactions, while simultaneously minimizing false-positive signals. Additionally, we conducted a sensitivity analysis in which adverse events associated with abrocitinib and upadacitinib were re-analyzed after excluding concomitant medications frequently used with JAK inhibitors. Infection and infestation-related AEs were considered positive signals if events met all screening criteria. All data processing and statistical analyses were performed using R (version 4.4.2) and Microsoft Excel (version 2021).

**Table 1:** Two-by-two contingency table for disproportionality analyses.

	Target AEs	Other AEs	Total
Target drug	a	b	a + b
Other drugs	c	d	c + d
Total	a + c	b + d	a + b + c + d

AEs, adverse events; a, the number of reports containing both the target drug and target adverse drug reaction; b, the number of reports containing other adverse drug reactions of the target drug; c, the number of reports containing the target adverse drug reaction of other drugs; d, the number of reports containing other drugs and other adverse drug reactions.

**Table 2:** Four major algorithms used for signal detection.

Algorithms	Equation	Criteria
ROR	ROR = ad/b/c	lower limit of 95% CI > 1, a ≥ 3
	95% CI = e^ln(ROR) ± 1.96(1/a + 1/b + 1/c + 1/d)^0.5^	
PRR	PRR = a(c + d)/c/(a + b)	PRR ≥ 2, χ^2^ ≥ 4, a ≥ 3
	χ^2^ = [(ad-bc)^2](a + b + c + d)/[(a + b)(c + d)(a + c)(b + d)]	
BCPNN	IC = log_2_a(a + b + c + d)(a + c)(a + b)	IC025 > 0
	95% CI = E(IC) ± 2 V(IC)^0.5	
MGPS	EBGM = a(a + b + c + d)/(a + c)/(a + b)	EBGM05 > 2
	95% CI = e^ln(EBGM) ± 1.96(1/a + 1/b + 1/c + 1/d)^0.5^	

a, the number of reports containing both the target drug and target adverse drug reaction; b, the number of reports containing other adverse drug reactions of the target drug; c, the number of reports containing the target adverse drug reaction of other drugs; and d, the number of reports containing other drugs and other adverse drug reactions. 95% CI, 95% confidence interval; N, the number of reports; χ2, chi-squared; IC, information component; IC025, the lower limit of 95% CI of the IC; E(IC), the IC expectations; V(IC), the variance of IC; EBGM, empirical Bayesian geometric mean; EBGM05, the lower limit of 95% CI of EBGM.

## 3 Results

### 3.1 Descriptive analysis

This study analyzed 298 reports associated with abrocitinib and 1,028 reports associated with upadacitinib. A higher proportion of male patients reported AEs for abrocitinib compared to female patients (55.4% vs. 39.9%), whereas the gender distribution for upadacitinib was more balanced (48.2% vs. 45.2%). For both drugs, the most frequently reported age group was 18 to 65 years (abrocitinib: 63.1%; upadacitinib: 41.1%). The number of reports consistently increased over time, with a notable increase in 2024 (abrocitinib: 138; upadacitinib: 433) compared to 2022 (abrocitinib: 48; upadacitinib: 139). The majority of reports for upadacitinib originated from healthcare professionals (57.47%), while those for abrocitinib were predominantly from non-healthcare sources (81.2%). Nearly all reports for both drugs were submitted from the United States. In terms of the severity, most reports for upadacitinib were classified as non-serious (62.5%), followed by hospitalization (35.0%), death (1.5%), and life-threatening events (1.1%). A similar pattern was observed for abrocitinib, with non-serious reports (63.8%) and hospitalization (29.9%) accounting for the majority of cases and with lower proportions of life-threatening events (9.4%), death (1.7%), and disability (1.3%) (Table 3).

**Table 3:** Clinical characteristics of infections and infestations related to JAK-1 inhibitors in atopic dermatitis treatment (Q3 201–Q1 2025).

Characteristics	Abrocitinib	Upadacitinib
Number of reports	298	1,028
Gender		
Female	165 (55.4%)	496 (48.2%)
Male	119 (39.9%)	465 (45.2%)
Missing	14 (4.7%)	67 (6.5%)
Age		
<18	28 (9.4%)	64 (6.2%)
18–65	188 (63.1%)	423 (41.1%)
>65	47 (15.8%)	175 (17.0%)
Missing	35 (11.7%)	366 (35.6%)
Outcomes		
Death	5 (1.7%)	15 (1.5%)
Disability	4 (1.3%)	0 (0.0%)
Hospitalization	89 (29.9%)	360 (35.0%)
Life-threatening	10 (9.4%)	11 (1.1%)
Other	190 (63.8%)	642 (62.5%)
Top 3 Reported Countries		
United States	123 (41.3%)	693 (67.4%)
Canada	66 (22.1%)	98 (9.5%)
Japan	25 (8.4%)	46 (4.5%)
Reporter		
Healthcare professional	49 (16.4%)	466 (45.3%)
Non-healthcare professional	242 (81.2%)	364 (35.4%)
Missing	7 (2.3%)	198 (19.3%)
Reporting year		
2019	0 (0.0%)	3 (0.3%)
2020	0 (0.0%)	9 (0.9%)
2021	0 (0.0%)	8 (0.8%)
2022	48 (16.1%)	139 (13.5%)
2023	66 (22.1%)	295 (28.7%)
2024	138 (46.3%)	433 (42.1%)
2025	46 (15.4%)	141 (13.7%)

### 3.2 Disproportionality analysis

**Figure 2:**
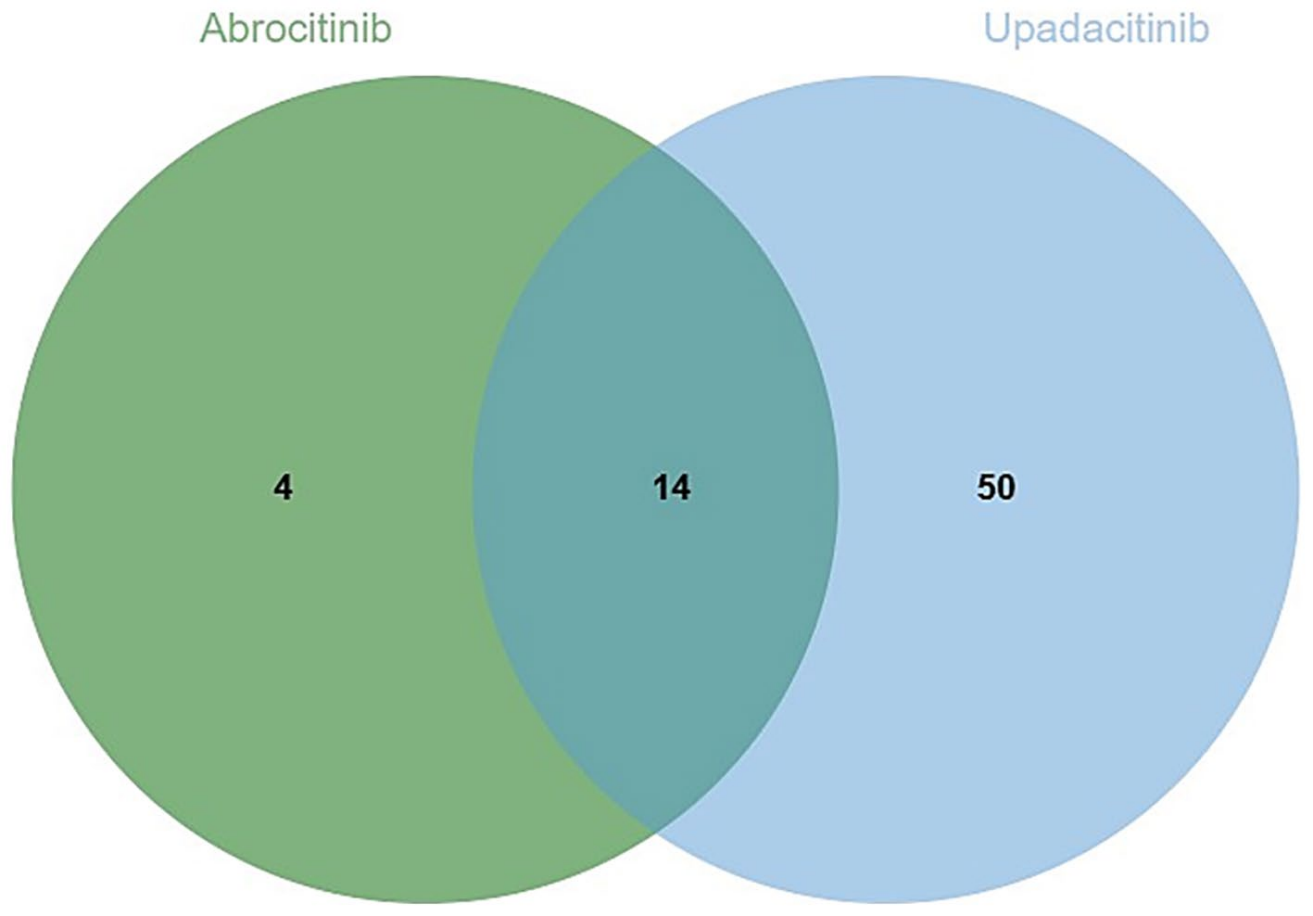
Venn diagram showing the types of AEs related to infection caused by two JAK1 inhibitors.

**Figure 3:**
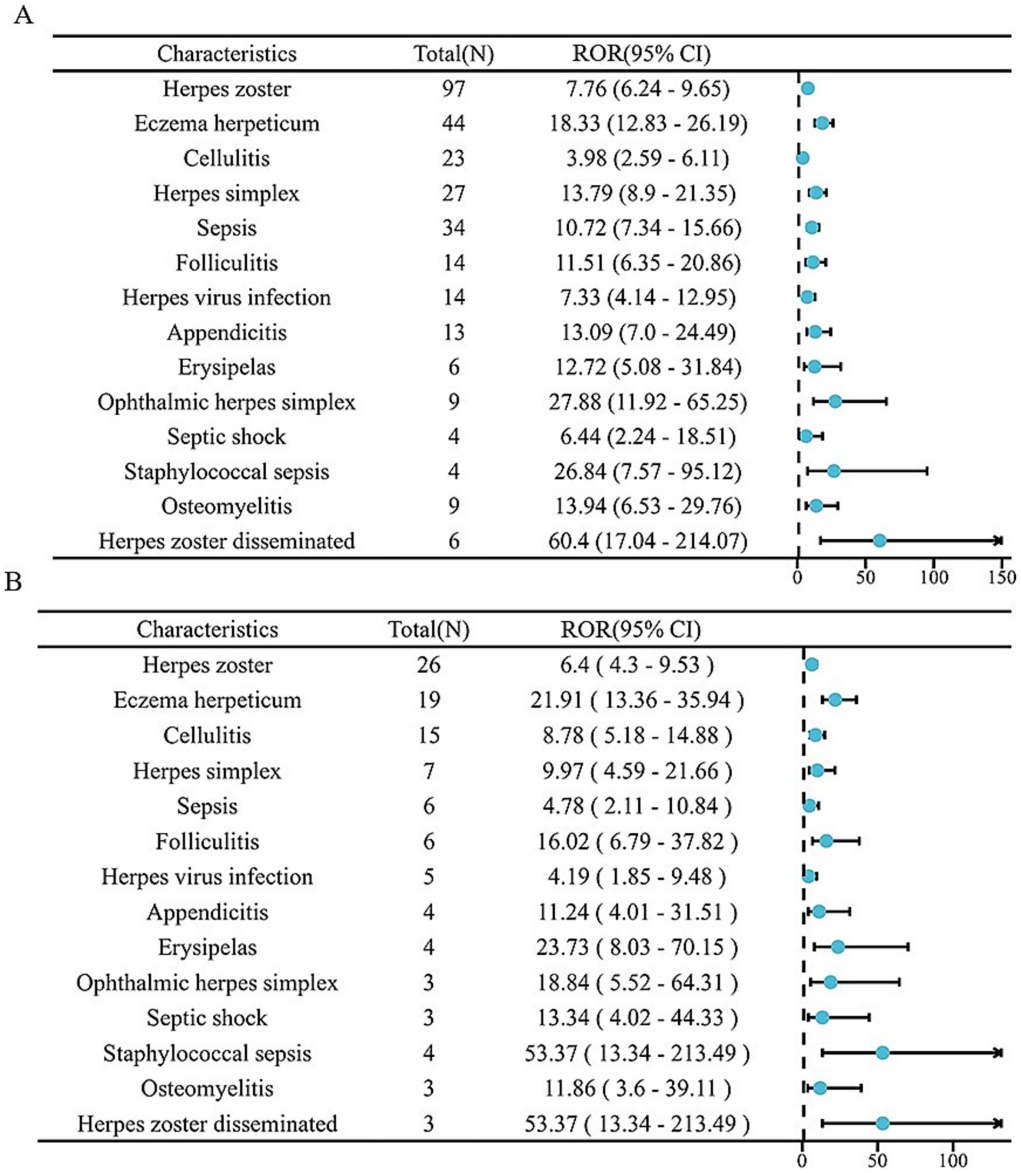
The forest plot of two drugs under the same PT conditions. **(A)** Upadacitinib; **(B)** Abrocitinib.

The analysis identified 18 positive signals associated with abrocitinib, among which the most frequently reported AEs included herpes zoster, eczema herpeticum, and herpes simplex. These findings confirm several previously recognized AEs, such as lower respiratory tract infections, herpes simplex, and folliculitis. Additionally, we detected several unexpected AEs, including sepsis, appendicitis, and septic shock, all of which yielded notable positive signals. For upadacitinib, a total of 64 positive AE reports were recorded. The most common events were herpes zoster, pneumonia, and influenza. Similarly, this investigation revealed unexpected adverse outcomes—such as sepsis, appendicitis, and septic shock among affected patients (Table 4). We found that there were 14 positive AEs that could be caused by both upadacitinib and abrocitinib, including herpes zoster, eczema herpeticum, herpes simplex, sepsis, appendicitis, septic shock (Figure 2). There were 4 positive AEs found only in abrocitinib, namely skin infection, impetigo, lower respiratory tract infection, and superinfection bacterial. Additionally, 50 adverse events were reported exclusively in patients treated with upadacitinib, such as pneumonia, influenza, and urinary tract infection (Supplementary Table S1). Furthermore, we compared the ROR signal strengths of the two drugs under identical PT conditions (Figure 3).

**Table 4:** Signal strength of positive infection and infestation-related adverse events associated with JAK-1 inhibitors in atopic dermatitis.

Biologics	PT	*N*	ROR (95% CI)	PRR (χ^2^)	EBGM (EBGM05)	IC (IC025)
Abrocitinib	Herpes zoster	26	6.4 (4.3–9.53)	6.36 (110.97)	6.06 (4.34)	2.6 (2.03)
	Eczema herpeticum	19	21.91 (13.36–35.94)	21.79 (312.99)	18.26 (12.07)	4.19 (3.49)
	Cellulitis	15	8.78 (5.18–14.88)	8.74 (95.09)	8.15 (5.24)	3.03 (2.28)
	Herpes simplex	7	9.97 (4.59–21.66)	9.95 (51.57)	9.19 (4.8)	3.2 (2.13)
	Sepsis	6	4.78 (2.11–10.84)	4.77 (17.15)	4.61 (2.33)	2.21 (1.09)
	Skin infection	6	4.19 (1.85–9.48)	4.18 (13.98)	4.06 (2.05)	2.02 (0.91)
	Folliculitis	6	16.02 (6.79–37.82)	16 (73.36)	14.04 (6.84)	3.81 (2.64)
	Impetigo	5	10.47 (4.18–26.25)	10.45 (38.94)	9.61 (4.45)	3.26 (2.03)
	Lower respiratory tract infection	5	10.27 (4.1–25.72)	10.25 (38.1)	9.44 (4.38)	3.24 (2)
	Herpes virus infection	5	9.05 (3.63–22.56)	9.04 (32.95)	8.41 (3.92)	3.07 (1.84)
	Appendicitis	4	11.24 (4.01–31.51)	11.22 (33.71)	10.25 (4.33)	3.36 (1.99)
	Erysipelas	4	23.73 (8.03–70.15)	23.7 (71.15)	19.57 (7.9)	4.29 (2.87)
	Ophthalmic herpes simplex	3	18.84 (5.52–64.31)	18.82 (43.02)	16.15 (5.78)	4.01 (2.44)
	Septic shock	3	13.34 (4.02–44.33)	13.33 (30.41)	11.96 (4.38)	3.58 (2.04)
	Staphylococcal sepsis	3	53.37 (13.34–213.49)	53.32 (102.68)	35.88 (11.25)	5.17 (3.46)
	Osteomyelitis	3	11.86 (3.6–39.11)	11.85 (26.82)	10.76 (3.97)	3.43 (1.89)
	Superinfection bacterial	3	40.03 (10.61–150.95)	39.99 (82.94)	29.36 (9.67)	4.88 (3.21)
	Herpes zoster disseminated	3	53.37 (13.34–213.49)	53.32 (102.68)	35.88 (11.25)	5.17 (3.46)
Upadacitinib	Herpes zoster	97	7.76 (6.24–9.65)	7.7 (475.31)	6.62 (5.52)	2.73 (2.41)
	Pneumonia	85	4.21 (3.36–5.27)	4.18 (186.8)	3.88 (3.22)	1.96 (1.63)
	Influenza	68	2.61 (2.04–3.33)	2.6 (62.86)	2.5 (2.03)	1.32 (0.96)
	Urinary tract infection	62	3.66 (2.82–4.75)	3.64 (109.18)	3.42 (2.75)	1.78 (1.4)
	Eczema herpeticum	44	18.33 (12.83–26.19)	18.26 (493.85)	12.87 (9.55)	3.69 (3.19)
	Sepsis	34	10.72 (7.34–15.66)	10.69 (236.05)	8.66 (6.31)	3.11 (2.58)
	Staphylococcal infection	33	5.64 (3.92–8.13)	5.63 (110.23)	5.06 (3.73)	2.34 (1.81)
	Herpes simplex	27	13.79 (8.9–21.35)	13.75 (238.05)	10.51 (7.29)	3.39 (2.78)
	Upper respiratory tract infection	24	3.3 (2.18–5.01)	3.3 (35.51)	3.12 (2.2)	1.64 (1.04)
	Cellulitis	23	3.98 (2.59–6.11)	3.97 (46.59)	3.71 (2.59)	1.89 (1.27)
	Diverticulitis	19	7.16 (4.39–11.66)	7.15 (85.32)	6.22 (4.13)	2.64 (1.94)
	Herpes virus infection	14	7.33 (4.14–12.95)	7.32 (64.62)	6.35 (3.94)	2.67 (1.86)
	Folliculitis	14	11.51 (6.35–20.86)	11.5 (104.39)	9.17 (5.57)	3.2 (2.37)
	Appendicitis	13	13.09 (7–24.49)	13.08 (109.47)	10.12 (5.99)	3.34 (2.47)
	Herpes ophthalmic	12	10.07 (5.35–18.96)	10.06 (78.35)	8.25 (4.86)	3.04 (2.16)
	Post-procedural infection	11	11.08 (5.68–21.6)	11.07 (79.02)	8.9 (5.09)	3.15 (2.23)
	Ophthalmic herpes zoster	11	24.62 (11.62–52.14)	24.59 (154.55)	15.64 (8.35)	3.97 (2.98)
	Cystitis	11	4.48 (2.4–8.35)	4.47 (26.69)	4.12 (2.45)	2.04 (1.17)
	Osteomyelitis	9	13.94 (6.53–29.76)	13.93 (80.25)	10.61 (5.62)	3.41 (2.37)
	Bacterial infection	9	4.47 (2.25–8.91)	4.47 (21.84)	4.12 (2.32)	2.04 (1.08)
	Ophthalmic herpes simplex	9	27.88 (11.92–65.25)	27.86 (137.73)	16.87 (8.28)	4.08 (2.98)
	Respiratory syncytial virus infection	8	4.54 (2.18–9.43)	4.53 (19.81)	4.18 (2.27)	2.06 (1.05)
	Arthritis infective	7	20.13 (8.12–49.9)	20.12 (84.81)	13.75 (6.43)	3.78 (2.59)
	Bacteremia	7	21.68 (8.65–54.36)	21.67 (89.71)	14.44 (6.69)	3.85 (2.65)
	Meningitis	7	20.13 (8.12–49.9)	20.12 (84.81)	13.75 (6.43)	3.78 (2.59)
	Erysipelas	6	12.72 (5.08–31.84)	12.71 (49.19)	9.9 (4.59)	3.31 (2.08)
	Latent tuberculosis	6	24.16 (8.78–66.49)	24.15 (83.21)	15.47 (6.63)	3.95 (2.65)
	Abscess	6	4.47 (1.92–10.4)	4.47 (14.56)	4.12 (2.04)	2.04 (0.89)
	Gastric infection	6	16.11 (6.25–41.52)	16.1 (60.69)	11.78 (5.34)	3.56 (2.31)
	Herpes zoster disseminated	6	60.4 (17.04–214.07)	60.37 (140.12)	24.75 (8.58)	4.63 (3.23)
	Gastroenteritis	6	12.72 (5.08–31.84)	12.71 (49.19)	9.9 (4.59)	3.31 (2.08)
	Herpes zoster cutaneous disseminated	5	100.66 (19.53–518.89)	100.61 (140.89)	29.46 (7.47)	4.88 (3.32)
	Wound infection	5	9.59 (3.61–25.43)	9.58 (31.04)	7.93 (3.51)	2.99 (1.69)
	Staphylococcal skin infection	5	12.58 (4.61–34.35)	12.58 (40.6)	9.82 (4.24)	3.3 (1.97)
	Skin bacterial infection	5	15.49 (5.52–43.45)	15.48 (48.91)	11.46 (4.83)	3.52 (2.17)
	Staphylococcal sepsis	4	26.84 (7.57–95.12)	26.83 (59.68)	16.5 (5.72)	4.04 (2.49)
	Device-related infection	4	13.42 (4.33–41.62)	13.41 (34.47)	10.31 (4)	3.37 (1.9)
	Tuberculosis	4	5.55 (1.95–15.8)	5.55 (13.12)	5 (2.08)	2.32 (0.94)
	Pulmonary tuberculosis	4	20.13 (6.06–66.86)	20.12 (48.46)	13.75 (5.04)	3.78 (2.26)
	Varicella	4	7.67 (2.63–22.34)	7.67 (19.48)	6.6 (2.7)	2.72 (1.31)
	Arthritis bacterial	4	14.64 (4.66–45.98)	14.63 (37.26)	11 (4.22)	3.46 (1.98)
	Abscess limb	4	9.47 (3.19–28.16)	9.47 (24.53)	7.86 (3.16)	2.97 (1.54)
	Otitis externa	4	17.89 (5.51–58.11)	17.89 (44.15)	12.69 (4.74)	3.67 (2.16)
	Vaginal infection	4	8.95 (3.03–26.44)	8.94 (23.09)	7.5 (3.03)	2.91 (1.48)
	Septic shock	4	6.44 (2.24–18.51)	6.44 (15.84)	5.69 (2.35)	2.51 (1.11)
	Urosepsis	4	23.01 (6.73–78.6)	23 (53.56)	15 (5.36)	3.91 (2.37)
	Beta hemolytic streptococcal infection	4	53.68 (12.01–239.88)	53.66 (88.59)	23.57 (6.73)	4.56 (2.91)
	Necrotizing fasciitis	4	40.26 (10.07–161)	40.24 (76.54)	20.62 (6.47)	4.37 (2.76)
	Tonsillitis	4	5.75 (2.02–16.4)	5.75 (13.73)	5.16 (2.15)	2.37 (0.98)
	Purulence	4	80.52 (14.75–439.66)	80.49 (104.67)	27.5 (6.64)	4.78 (3.09)
	Pyelonephritis	3	7.55 (2.2–25.91)	7.55 (14.35)	6.51 (2.32)	2.7 (1.12)
	Pneumocystis jirovecii pneumonia	3	15.1 (4–56.91)	15.09 (28.71)	11.25 (3.71)	3.49 (1.83)
	Dermatitis infected	3	10.06 (2.84–35.67)	10.06 (19.59)	8.25 (2.86)	3.04 (1.43)
	Peritonsillar abscess	3	24.15 (5.77–101.08)	24.15 (41.6)	15.47 (4.67)	3.95 (2.22)
	Herpes simplex reactivation	3	120.77 (12.56–1161.14)	120.73 (89.06)	30.93 (4.66)	4.95 (3.02)
	Hepatitis e	3	60.38 (10.09–361.42)	60.37 (70.06)	24.75 (5.54)	4.63 (2.77)
	Oral pustule	3	120.77 (12.56–1161.14)	120.73 (89.06)	30.93 (4.66)	4.95 (3.02)
	Dengue fever	3	60.38 (10.09–361.42)	60.37 (70.06)	24.75 (5.54)	4.63 (2.77)
	Acne pustular	3	15.1 (4–56.91)	15.09 (28.71)	11.25 (3.71)	3.49 (1.83)
	Wound infection bacterial	3	120.77 (12.56–1161.14)	120.73 (89.06)	30.93 (4.66)	4.95 (3.02)
	Body tinea	3	12.08 (3.32–43.89)	12.07 (23.44)	9.52 (3.23)	3.25 (1.61)
	Epstein–Barr virus infection	3	24.15 (5.77–101.08)	24.15 (41.6)	15.47 (4.67)	3.95 (2.22)
	Staphylococcal bacteremia	3	6.36 (1.88–21.48)	6.35 (11.69)	5.62 (2.03)	2.49 (0.93)
	Eczema infected	3	7.1 (2.08–24.24)	7.1 (13.37)	6.19 (2.21)	2.63 (1.05)

### 3.3 Sensitivity analysis

We excluded reports in which the abrocitinib or upadacitinib were concomitantly used with the medications most commonly prescribed for the management of AD, including commonly used topical agents such as betamethasone valerate, beclometasone, tacrolimus, pimecrolimus, mupirocin, oral antihistamines such as desloratadine and levocetirizine, and, in rare cases, the biologic agent dupilumab. After excluding the combined use of the above drugs, we identified 15 positive signals with abrocitinib and 40 positive signals with upadacitinib. Persistent potential adverse reactions included herpes zoster, eczema herpeticum, and herpes simplex. Sepsis, appendicitis, and septic shock (Supplementary Table S2).

## 4 Discussion

In recent years, advances in understanding the pathogenesis of AD have led to the introduction of oral JAK inhibitors as a novel treatment strategy in clinical practice (17). This study provided a comprehensive evaluation of AEs associated with upadacitinib and abrocitinib following their market approval in 2022 using data from the FAERS database. We confirmed several previously recognized AEs related to oral JAK-1 inhibitors in AD treatment, including herpes zoster, eczema herpeticum, and influenza. Additionally, we identified several unlabeled AEs such as sepsis, appendicitis, and septic shock.

Our findings indicate that the highest number of reported AEs for both upadacitinib and abrocitinib in AD treatment were related to herpes zoster. Typically, herpes zoster presents with clustered vesicles in a unilateral dermatomal distribution accompanied by severe pain (18). These symptoms significantly impair patients’ daily functioning (19), and postherpetic neuralgia can profoundly affect physical and mental health, as well as the quality of life (20). Multiple clinical trials have established herpes zoster as a serious adverse reaction associated with oral JAK inhibitors in the treatment of AD. For example, one clinical trial reported herpes zoster incidence rates of 0.5 and 0.2% for abrocitinib at 200 mg and 100 mg, respectively (21). A meta-analysis of 16 clinical trials involving 10,689 patients showed that upadacitinib was associated with a dose-dependent increase in herpes zoster incidence at dosages of 15 mg and 30 mg compared to placebo (22). The Janus kinase-dependent immune function is involved in numerous steps in the viral life cycle pathway of the varicella-zoster virus. Inhibiting these JAK-dependent pathways may increase susceptibility to herpes zoster (23). Our research findings are consistent with previous studies. The real-world study by van der Gang LF et al. found that both upadacitinib and abrocitinib had herpes virus infections as the most frequently occurring AEs, which is consistent with the infection pattern reported in moderate to severe AD (12). Similarly, Tsai et al. (10) used US healthcare data and observed that JAK inhibitor treatment for AD increased the risk of herpes zoster compared to the dupilumab group (HR 2.51, 95% CI: 1.14–5.52). Furthermore, multiple factors have been reported to collectively contribute to an increased susceptibility to herpes zoster. These include advanced age (particularly > 65 years), a personal or family history of herpes zoster, female sex, Asian and Oceanian ethnicity, and concomitant use of immunosuppressive drugs (such as glucocorticoids), as well as smoking, alcohol consumption, and psychological stress (24, 25). Therefore, risk assessment and vigilant monitoring of patients with these risk factors are crucial in clinical practice. The recombinant herpes zoster vaccines have been shown to effectively and safely reduce the incidence and severity of herpes zoster. When appropriate, vaccination should be considered for AD patients receiving JAK inhibitors (26).

Another notable AE was herpes simplex, which commonly presents as vesicular lesions, ulcers, pain, and pruritus on the lips or genitalia (25). Consistent with our findings, a 2-year observational study found that oral JAK inhibitor treatment for AD increased the risk of herpes simplex infection (HR 2.26, 95% CI 1.36–3.74; BH-adjusted *p* = 0.004) (10). In a phase III clinical trial of abrocitinib, the incidence of herpes simplex was 6.1% at the 100 mg dose (26). Recurrent herpes simplex may increase the likelihood of treatment discontinuation. Furthermore, herpes simplex in AD patients may progress to eczema herpeticum, a disseminated infection that may become life-threatening if not managed promptly (27). A meta-analysis reported a 2.3% incidence of eczema herpeticum with abrocitinib 100 mg (21). Our study also identified cases of eczema herpeticum in patients treated with either abrocitinib or upadacitinib as the second most frequently reported AE for abrocitinib. These findings reinforce the need for clinicians to maintain high vigilance for herpes simplex and eczema herpeticum during JAK inhibitor therapy. Proactive education, regular monitoring, and early intervention are crucial for reducing risks.

Moreover, our analysis revealed several AEs not currently included on drug labels, most notably sepsis. Both abrocitinib and upadacitinib were associated with sepsis across four analytical methods, although the underlying mechanism remains unclear. Tsai et al. (10) also found that adverse events of sepsis occurred in AD patients treated with either JAK inhibitors or dupilumab, but there was no statistical difference between the two (*p* = 0.065 > 0.05). The JAK–STAT pathway plays complex and context-dependent roles in sepsis pathogenesis, and its modulation may have divergent effects at different stages of the condition (27). The precise mechanisms by which JAK inhibitors may induce or exacerbate sepsis remain incompletely understood. Sepsis presents as a systemic inflammatory response with an estimated mortality rate of 30–40% (28). Clinicians should remain vigilant, particularly in high-risk patients such as the elderly, particularly those with diabetes or neutropenia, even without warning labels. Active infections should be ruled out before initiating treatment. Upon systemic infection during treatment, drug discontinuation and anti-infective therapy are required.

Appendicitis was identified as another unlabeled AE associated with this medication. Typically, the condition presents with pain that originates around the umbilicus and subsequently migrates to the lower right quadrant of the abdomen. If surgical intervention for appendicitis is not undertaken promptly (ideally within 24 h), a risk of severe complications such as peritonitis, sepsis, or septic shock exists (29). Abdominal pain is a known side effect of JAK inhibitors (30). However, the anti-inflammatory properties of these drugs may mask classic appendicitis symptoms, delaying diagnosis and increasing the risk of perforation (31). Therefore, new-onset abdominal pain during treatment warrants immediate clinical evaluation to weigh the therapeutic benefits against the potential risk of gastrointestinal events.

Although both upadacitinib and abrocitinib are selective JAK-1 inhibitors, differences exist between them. Evidence from studies in healthy volunteers indicates that upadacitinib administration inhibits IL-6 (via the JAK-1/JAK-2 pathway)-induced STAT3 phosphorylation and IL-7 (via the JAK-1/JAK-3 pathway)-induced STAT5 phosphorylation in whole blood samples in a dose- and concentration-dependent manner (32). In contrast, abrocitinib inhibits multiple cytokine signaling pathways that play key roles in the pathogenesis of AD, including IL-4, IL-13, IL-31, and IFN-γ (33). Our study identified herpes zoster as the most common and well-defined infection risk associated with both drugs. Beyond herpes zoster, the most frequently reported events for upadacitinib included pneumonia and influenza, whereas for abrocitinib, they included eczema herpeticum and herpes simplex. This may suggest that, in clinical practice, upadacitinib is associated with more reports of respiratory infections, while abrocitinib may be more notably linked to specific cutaneous viral infections, though further research is required for confirmation. Positive signals were detected for both drugs regarding sepsis, septic shock, and appendicitis. These represent potentially serious risks not sufficiently emphasized in previous labels, warranting high clinical vigilance. Currently, no head-to-head studies directly comparing the efficacy and safety of upadacitinib and abrocitinib in AD are available. A recent network meta-analysis of phase 3 clinical trials suggested that, at 12–16 weeks, upadacitinib 30 mg was the most effective systemic therapy, followed by abrocitinib 200 mg and upadacitinib 15 mg (34). Some studies have reported good responses to abrocitinib following an inadequate response to upadacitinib in AD treatment (35). Patients taking oral JAK inhibitors require baseline and periodic laboratory monitoring, including complete blood count with differential, liver function tests, lipid profiles, and screening for tuberculosis, hepatitis B, and hepatitis C (36). Research by van der Gang et al. identified a history of infection, particularly viral or fungal skin infections, as an independent risk factor associated with infection, indicating that this should be carefully evaluated when considering JAK inhibitor therapy (12). Based on our findings, we suggest that, for patients with a history of recurrent respiratory infections or high risk, abrocitinib may be prioritized (given its relatively lower reporting of pneumonia and influenza in this study), while remaining vigilant for all infection risks. For patients with a history of severe or frequent herpes viral infections (especially eczema herpeticum), extreme caution is warranted. Our study suggests a stronger association of abrocitinib with this event, although both drugs carry risks. Prophylactic strategies should be considered before initiation, accompanied by close monitoring. Treatment selection is determined by multiple factors, including safety, risk factors, comorbidities, and cost. Future large-scale, prospective head-to-head studies with more data and longer follow-up are needed to analyze the efficacy and safety of these two JAK-1 inhibitors in AD.

This study utilized data mining techniques on the FAERS database to identify previously unreported infection-related signals and support safer clinical use. Several limitations should be acknowledged. First, as a spontaneous reporting system, FAERS is subject to reporting biases due to its voluntary nature; the study can only infer statistical associations between the drugs and adverse events, rather than causality. Second, the FAERS database only records the AE events that have occurred, lacking a control group, and thus cannot directly assess infection risks or increased incidence of infection. Additionally, the database often lacks detailed information on drug dosage, treatment duration, concomitant medications, and patient comorbidities, limiting a deeper analysis of risk factors. Finally, given the nature of real-world data, analyzing single-drug exposure reports is difficult. Our analysis only excluded reports where abrocitinib or upadacitinib were used concomitantly with the most common AD therapies. Therefore, in the future, larger-scale prospective studies with more data and longer follow-up periods will be needed to further assess the safety of JAK-1 inhibitors in AD.

## 5 Conclusion

While most reported AEs aligned with known risks described in the product labeling, we also identified potential unlabeled signals such as sepsis and appendicitis. These findings underscore the importance of vigilant monitoring and risk mitigation in patients receiving abrocitinib or upadacitinib, contributing to safer clinical use of these agents.

## Data Availability

The original contributions presented in the study are included in the article/Supplementary material, further inquiries can be directed to the corresponding author.
