# Supplementary material for: Association between oral JAK-1 inhibitors and infection risks in atopic dermatitis: a retrospective analysis of the FAERS database
Source: Front Med (Lausanne). 2025 Nov 3;12:1694688. doi: 10.3389/fmed.2025.1694688 (PMC12620211; doi:10.3389/fmed.2025.1694688)
Supplement: Supplementary file 1 [file Data_Sheet_1.PDF]

Table S1: Positive Signals Exclusively Associated with Abrocitinib, Upadacitinib, or Both

abrocitinib	Both	upadacitinib	
Skin infection,	Herpes zoster	Pneumonia	Pulmonary tuberculosis
Impetigo	Eczema herpeticum	Influenza	Varicella
Lower respiratory tract infection,	Cellulitis	Urinary tract infection	Arthritis bacterial
Superinfection bacterial	Herpes simplex	Staphylococcal infection	Abscess limb
	Sepsis	Upper respiratory tract infection	Otitis externa
	Folliculitis	Diverticulitis	Vaginal infection
	Herpes virus infection	Herpes ophthalmic	Urosepsis
	Appendicitis	Post procedural infection	Beta haemolytic streptococcal infection
	Erysipelas	Ophthalmic herpes zoster	Necrotising fasciitis
	Ophthalmic herpes simplex	Cystitis	Tonsillitis
	Septic shock	Bacterial infection	Purulence
	Staphylococcal sepsis	Respiratory syncytial virus infection	Pyelonephritis
	Osteomyelitis	Arthritis infective	Pneumocystis jirovecii pneumonia
	Herpes zoster disseminated	Bacteraemia	Dermatitis infected
		Meningitis	Peritonsillar abscess
		Latent tuberculosis	Herpes simplex reactivation
		Abscess	Hepatitis e
		Gastric infection	Oral pustule
		Gastroenteritis	Dengue fever
		Herpes zoster	Acne pustular

		cutaneous disseminated	
		Wound infection	Wound infection bacterial
		Staphylococcal skin infection	Body tinea
		Skin bacterial infection	Epstein-barr virus infection
		Device related infection	Staphylococcal bacteraemia
		Tuberculosis	Eczema infected

Table S2: Signal Strength of Positive Infection and Infestation-Related Adverse Events Associated with JAK-1 Inhibitors in Atopic Dermatitis excluding common medication co-usage at the PT level from FAERS data

Biologics	PT	N	ROR(95%CI)	PRR(χ^2)	EBGM(EBGM05)	IC(IC025)
Abrocitinib	Herpes Zoster	23	6.48 (4.25 - 9.88)	6.44 (100.31)	6.16 (4.04)	2.62 (1.73)
	Eczema Herpeticum	14	17.09 (9.75 - 29.97)	17.01 (184.4)	14.99 (8.55)	3.91 (2.15)
	Cellulitis	13	8.74 (4.97 - 15.37)	8.7 (82.6)	8.17 (4.65)	3.03 (1.63)
	Herpes Simplex	7	11.33 (5.21 - 24.62)	11.3 (59.98)	10.4 (4.78)	3.38 (1.18)
	Sepsis	6	5.37 (2.37 - 12.17)	5.36 (20.34)	5.17 (2.28)	2.37 (0.57)
	Skin Infection	6	4.85 (2.14 - 10.99)	4.84 (17.58)	4.69 (2.07)	2.23 (0.5)
	Folliculitis	5	14.4 (5.68 - 36.46)	14.37 (55.45)	12.92 (5.1)	3.69 (0.85)
	Impetigo	4	10.26 (3.69 - 28.52)	10.25 (30.71)	9.51 (3.42)	3.25 (0.45)
	Appendicitis	4	12.76 (4.54 - 35.82)	12.74 (39.06)	11.59 (4.13)	3.54 (0.52)
	Herpes Virus Infection	4	8.28 (3 - 22.84)	8.27 (23.89)	7.79 (2.83)	2.96 (0.37)
	Erysipelas	3	22.12 (6.44 - 75.96)	22.1 (50.89)	18.76 (5.46)	4.23 (0.19)
	Osteomyelitis	3	13.11 (3.97 - 43.23)	13.09 (30.16)	11.88 (3.6)	3.57 (0.13)
	Septic Shock	3	14.75 (4.44 - 49)	14.73 (34.13)	13.2 (3.97)	3.72 (0.15)
	Staphylococcal Sepsis	3	70.78 (16.91 - 296.34)	70.71 (128.85)	44.57 (10.65)	5.48 (0.16)
	Herpes Zoster Disseminated	3	58.99 (14.74 - 235.98)	58.92 (113.88)	39.61 (9.9)	5.31 (0.18)
Upadacitinib	Herpes Zoster	90	7.7 (6.15 - 9.65)	7.64 (440.02)	6.62 (5.28)	2.73 (2.31)
	Pneumonia	83	4.47 (3.56 - 5.61)	4.44 (200.45)	4.11 (3.28)	2.04 (1.65)
	Influenza	66	2.78 (2.16 - 3.56)	2.76 (69.91)	2.66 (2.07)	1.41 (1.01)
	Urinary Tract Infection	61	3.99 (3.06 - 5.19)	3.97 (123.98)	3.71 (2.85)	1.89 (1.45)
	Eczema Herpeticum	40	17.07 (11.82 - 24.66)	17.01 (429.32)	12.4 (8.58)	3.63 (2.76)
	Sepsis	33	10.97 (7.48 - 16.1)	10.94 (236.57)	8.89 (6.06)	3.15 (2.3)
	Staphylococcal Infection	31	5.79 (3.97 - 8.43)	5.77 (107.65)	5.2 (3.57)	2.38 (1.65)
	Herpes Simplex	24	12.66 (8.02 - 19.98)	12.63 (197.7)	9.94 (6.3)	3.31 (2.22)
	Upper Respiratory Tract Infection	23	3.46 (2.26 - 5.3)	3.46 (37.15)	3.27 (2.14)	1.71 (0.96)
	Cellulitis	20	3.71 (2.35 - 5.87)	3.71 (36.38)	3.49 (2.21)	1.8 (0.98)
	Diverticulitis	19	7.78 (4.77 - 12.69)	7.76 (94.55)	6.71 (4.11)	2.75 (1.68)
	Folliculitis	14	12.04 (6.65 - 21.82)	12.03 (110.09)	9.58 (5.29)	3.26 (1.77)
	Herpes Virus Infection	14	7.97 (4.5 - 14.12)	7.96 (71.69)	6.86 (3.87)	2.78 (1.48)
	Cystitis	11	4.93 (2.64 - 9.21)	4.93 (30.81)	4.51 (2.42)	2.17 (0.91)
	Appendicitis	11	11.3 (5.81 - 22)	11.29 (81.37)	9.12 (4.68)	3.19 (1.5)
	Herpes Ophthalmic	11	9.66 (5.01 - 18.6)	9.65 (69.36)	8.03 (4.17)	3.01 (1.41)
	Post Procedural Infection	11	12.2 (6.23 - 23.87)	12.18 (87.58)	9.67 (4.94)	3.27 (1.55)
	Ophthalmic Herpes Zoster	10	23.41 (10.8 - 50.72)	23.38 (137.75)	15.39 (7.1)	3.94 (1.69)
	Ophthalmic Herpes	9	31.6 (13.31 - 75)	31.57 (152.23)	18.47 (7.78)	4.21 (1.63)

Simplex					
Meningitis	7	21.06 (8.5 - 52.19)	21.04 (89.1)	14.36 (5.8)	3.84 (1.21)
Osteomyelitis	7	10.53 (4.6 - 24.11)	10.52 (48.26)	8.62 (3.76)	3.11 (1)
Bacteraemia	6	21.06 (7.9 - 56.12)	21.04 (76.37)	14.36 (5.39)	3.84 (1)
Latent Tuberculosis	6	25.27 (9.18 - 69.54)	25.25 (87.35)	16.16 (5.87)	4.01 (1.03)
Gastric Infection	6	18.05 (6.93 - 46.98)	18.04 (67.6)	12.93 (4.97)	3.69 (0.97)
Arthritis Infective	6	18.05 (6.93 - 46.98)	18.04 (67.6)	12.93 (4.97)	3.69 (0.97)
Herpes Zoster					
Disseminated	6	63.17 (17.82 - 223.9)	63.13 (146.76)	25.85 (7.29)	4.69 (1.08)
Gastroenteritis	5	11.08 (4.14 - 29.68)	11.08 (36.29)	8.98 (3.35)	3.17 (0.61)
Wound Infection	5	10.53 (3.95 - 28.06)	10.52 (34.47)	8.62 (3.23)	3.11 (0.6)
Erysipelas	5	12.39 (4.57 - 33.58)	12.38 (40.42)	9.79 (3.61)	3.29 (0.64)
Staphylococcal Skin					
Infection	5	13.16 (4.82 - 35.93)	13.15 (42.78)	10.26 (3.76)	3.36 (0.66)
Varicella	4	8.02 (2.75 - 23.37)	8.02 (20.64)	6.89 (2.37)	2.79 (0.23)
Otitis Externa	4	18.71 (5.76 - 60.78)	18.71 (46.41)	13.26 (4.08)	3.73 (0.41)
Septic Shock	4	6.74 (2.34 - 19.36)	6.73 (16.84)	5.94 (2.07)	2.57 (0.17)
Abscess Limb	4	9.91 (3.33 - 29.45)	9.9 (25.92)	8.21 (2.76)	3.04 (0.3)
Urosepsis	4	24.06 (7.04 - 82.21)	24.05 (56.24)	15.67 (4.59)	3.97 (0.43)
Pulmonary Tuberculosis	4	21.05 (6.34 - 69.93)	21.04 (50.91)	14.36 (4.32)	3.84 (0.42)
Arthritis Bacterial	4	18.71 (5.76 - 60.78)	18.71 (46.41)	13.26 (4.08)	3.73 (0.41)
Device Related Infection	4	15.31 (4.87 - 48.09)	15.31 (39.22)	11.49 (3.66)	3.52 (0.39)
Necrotising Fasciitis	4	42.11 (10.53 - 168.39)	42.09 (80.23)	21.54 (5.39)	4.43 (0.44)
Skin Bacterial Infection	4	14.04 (4.53 - 43.53)	14.03 (36.3)	10.77 (3.47)	3.43 (0.37)
